# Analysis of Sera of Recipients with Allograft Rejection Indicates That Keratin 1 Is the Target of Anti-Endothelial Antibodies

**DOI:** 10.1155/2017/8679841

**Published:** 2017-02-07

**Authors:** Xuli Guo, Juan Hu, Weiguang Luo, Qizhi Luo, Jing Guo, Fang Tian, Yingzi Ming, Yizhou Zou

**Affiliations:** ^1^Department of Immunology, Xiangya School of Medicine, Central South University, Hunan 410008, China; ^2^Research Laboratory of Blood Transfusion, Blood Center of Guizhou Province, Guizhou 550002, China; ^3^Transplantation Center, The Third Xiangya Hospital of Central South University, Hunan 410013, China; ^4^The Cooperative Innovation Center of Engineering and New Products for Developmental Biology of Hunan Province, Hunan 410006, China

## Abstract

Anti-endothelial cell antibodies (AECAs) are usually directed against the surface antigens on the vascular endothelial cells. Clinical studies suggest a pathogenic role for nonhuman leukocyte antigen in antibody-mediated rejection; however, the antigens on the donor vascular endothelium that serve as the first-line targets for an immune response during allograft rejection have not been fully identified. Here, we used immunoprecipitation and mass spectrometry to identify antigens from the sera of kidney transplant recipients who were experiencing antibody-mediated rejection. Keratin 1 (KRT1) was identified as a novel antigenic target expressed on endothelial cells. To validate our finding, we produced recombinant proteins representing the three most common alleles of KRT1. The serum used for immunoprecipitation showed a strong reaction to KRT1 recombinants in western blot and ELISA. In the kidney transplant cohort, more AECA-positive recipients than AECA-negative recipients had KRT1 antibodies (32.2% versus 11.9%, *p* = 0.002). Sera from 255 renal recipients were tested by ELISA. Of the 77 recipients with deteriorating graft function (serum creatinine > 120 *μ*mol/L), 23 had anti-KRT1 antibodies. KRT1-IgG positivity was, therefore, associated with a higher risk of kidney transplant rejection (29.9% (23/77) versus 16.9% (30/178), *p* = 0.0187). A better understanding of this antigenic target will improve long-term allograft survival.

## 1. Introduction

Despite progress in matching donors with recipients, organ transplant rejection remains a barrier to successful transplantation. The human leukocyte antigens (HLA) are targets of the immune response against the donor tissue; however, rejection occurs in kidney allografts from HLA-identical siblings [1] and in the absence of donor-specific antibodies against the HLA antigens [2]. Thus, non-HLA antigens expressed in the graft endothelium, and not normally detectable on peripheral blood lymphocytes, must be involved in transplant rejection. It has been reported that antibodies against non-HLA antigens such as MICA [3–5], vimentin [6], tubulin [7, 8], myosin [9], collagen [8, 10], and angiotensin II type 1 receptor (AT1R) [11–13] may interfere with allograft. Vascular endothelium of graft is the first line of contact with the blood circulation and this primary site bears a host immune attack. The unexplained rejection occurred in the organ transplantation with negative-lymphocyte-crossmatches, suggesting that anti-endothelial cell antibodies (AECAs) [14–18] are a cause of antibody-mediated rejection (AMR) [19].

Non-HLA antigens expressed on donor allograft endothelial cells are of particular interest given that the vascular endothelium of a donated organ comes into physical contact with the recipient's immune system. Jackson and colleagues provided evidence for the clinic relevance of AECAs in kidney allograft rejection by analysis of donor-derived endothelial cell precursors [20]. Although the endothelial cell crossmatch (XM-ONE) has been shown to be clinically useful [21, 22], antigens expressed on endothelial cells make this assay technically challenging to implement. Identification of the exactly antigenic targets on endothelial cells would be able to develop the solid-phase immunoassays for the pretransplant risk assessment.

Several target molecules of AECAs were identified using endothelial cells and posttransplant sera from kidney and heart allograft recipients undergoing rejection in our previous investigation [18] and others [23]. Among these identified AECA-targeting proteins, Keratin 1 (KRT1) become more interesting because of its gene polymorphism [24] and it appears expressed on the surface of endothelial cells [25].

In the present study we established a more efficient approach to isolate and purify the specific IgG antibodies targeting vascular endothelium antigens using serum samples from the recipients under renal transplant rejection. KRT1 as the target protein was frequently identified in our experiments with immunoprecipitation and the mass spectrometry. In order to investigate the clinic impact of KRT1 antibodies in organ transplantation, three KRT1 recombinant proteins encoded by three common KRT1 alleles were produced for the antibody-detection assay. In this article we first report the characterization of KRT1 antibodies in kidney transplant patients and the association of anti-KRT1 antibodies with the outcome of allograft function in clinic.

## 2. Materials and Methods

### 2.1. Serum Specimens and DNA Samples

Sera were collected from 255 kidney transplant recipients during follow-up from 2012 to 2016. The front 160 sera were tested for AECAs with no-donor random HUVEC follow cytometry. Five sera were selected for antibody identification from transplant recipients who had received kidney allografts and undergoing rejection. Sera selected met the following criteria: (1) serum creatinine level > 400 *μ*mol/L, (2) random human umbilical cord vein endothelial cell (HUVEC) flow crossmatch with positive reaction, (3) anti-HLA or MICA antibody positive or HUVEC crossmatch positive, and (4) C4d positive. Collection of clinic samples and the research protocol was approved by the Ethics Committee of the 3rd Hospital of Xiangya Medical School (2014-S091). All participants provided written informed consent. One serum (S5) which only contains anti-EC was used for immunoprecipitation and determination of target antigen. Normal human sera (NHS) were obtained from volunteers through a protocol approved by the Institutional Review Board of the 3rd Hospital of Xiangya. DNA was extracted from leucocytes and purified using QIAamp DNA Blood Mini Kits (Qiagen, Valencia, CA, USA).

### 2.2. HUVEC Isolation and Flow Cytometry

Human umbilical cords (*N* = 8) and cord blood were obtained from Hunan Provincial Maternity and Child Care Hospital (HPMCCH) following a protocol approved by HPMCCH and Xiangya School of Medicine of Central South University Institutional Review Boards. HUVECs were obtained as the previous procedure [26]; in brief, umbilical cord veins were cannulated, washed with phosphate buffered saline (PBS) solution, and treated with collagenase I (0.2% in PBS) at 37°C for 20 min. Endothelial cells were collected and cultured in EBM-2 medium (Lonza, Walkersville, MD, USA) with 10% fetal bovine serum (FBS, Gibco, Grand Island, NY, USA) for 3–5 days. Cultured cells were washed with PBS, digested with 0.25% trypsin, and used for flow cytometry assays. To confirm HUVEC identity, after two washes, the cells were stained with PE-conjugated mouse anti-human CD31 (BD Biosciences, San Jose, CA, USA) and incubated at room temperature for 30 min. For AECA screening, 6 × 10^5^ cells were mixed with 30 *μ*L of undiluted serum and incubated at room temperature for 30 min. Pretitrated FITC-coupled goat anti-human IgG (BD Biosciences) was added after three washes. Cells were analyzed using a Gallios flow cytometer (Beckman, Brea, CA, USA).

### 2.3. Cord Blood Mononuclear Cell Isolation and Flow Cytometry

Anticoagulant-treated cord blood samples were diluted with an equal volume of PBS and gently added and centrifuged at 800*g* for 25 min. The mononuclear cells were isolated and washed with PBS three times. 3 × 10^5^ cells were mixed with 30 *μ*L of undiluted test serum and incubated at room temperature for 30 min. After three washes with PBS, pretitrated FITC-coupled goat anti-human IgG (BD Biosciences) and PE-coupled anti-CD3 (BD Biosciences) were added. CD3 facilitated T cell gating. Normal human sera were used as controls. Cells were analyzed using a Gallios flow cytometer (Beckman). The results were analyzed using FlowJo.

### 2.4. Detection of Anti-HLA and Anti-MICA Antibodies with Single Antigen Bead Array Flow Cytometry

IgG antibodies against HLA class I (A, B, and C) and class II (DR, DQ, and DP) were detected using a single antigen Luminex flow cytometry (Immucor) according to the protocol suggested by the manufacturer. MICA antibody testing was performed on patient serum samples using single antigen beads conjugated with recombinant MICA^*∗*^001, ^*∗*^002, ^*∗*^004, ^*∗*^007, ^*∗*^008, ^*∗*^009, ^*∗*^012, ^*∗*^016, ^*∗*^017, ^*∗*^018, ^*∗*^019, and ^*∗*^045. This kit was prepared in our laboratory and validated using the reference sera obtained from the 16th International Histocompatibility and Immunogenetics MICA workshop [27]. Antibody specificity was based on normalized mean fluorescence intensity (MFI) greater than 2000.

### 2.5. Antibody Absorption and Elution

Selected sera with antibodies against endothelial cells were obtained from five kidney allograft recipients. For absorption of antibodies, 1.0 × 10^7^ HUVECs were harvested and washed with PBS three times. Fifty microliters of serum was added to washed HUVECs; samples were incubated on ice for 1 h. When the purpose was to remove specific antibodies from serum, absorptions were performed two to four times with fresh HUVECs until no detectable antibodies remained. When the purpose was to obtain specific antibodies bound to HUVECs after serum absorption, cells were washed with PBS three times, and the bound antibodies were eluted by adding 45 *μ*L of elution buffer (0.13 M citric acid, 60 mM Na_2_HPO_4_, pH 3.0). Eluates were immediately neutralized by addition of 5 *μ*L of 1 M NaH_2_PO_4_ (pH 9.0).

### 2.6. Immunoprecipitation

Immunoprecipitations were carried out using the Direct Immunoprecipitation Kit (Pierce, Rockford, IL, USA) according to the manufacturer's instructions. Selected serum samples were incubated with 5 × 10^4^ fresh HUVEC cells at 4°C for 2 h. Cells were washed three times with PBS. The cell pellets were lysed in lysis buffer (Invitrogen) on ice for 30 min. The lysate was coupled to the protein A/G magnet beads and incubated with gentle rotation overnight at 4°C. The beads were washed four times with 0.1 M PBS, pH 8.0. Saline citrate buffer (0.1 M trisodium citrate, pH 3.0) was added to elute the antigen-antibody complex.

### 2.7. Western Blotting

Proteins were incubated at 95–100°C for 10 min and then analyzed on a 10% SDS-PAGE gel. After the proteins were transferred from the gel to an immunoblot polyvinylidene difluoride (PVDF, Sigma) membrane, the proteins were blocked with 5% fat-free milk solution in 0.05% Tween-20 PBS. The monoclonal antibodies HC10 and 1.7AD [28, 29], rabbit serum, or isolated and purified serum antibodies were used for western blot assays. After three washes, horseradish peroxidase (HRP) conjugated goat anti-mouse, anti-rabbit, or anti-human IgG secondary antibodies (Jackson Laboratories, Bar Harbor, ME, USA) were added. Chemiluminescent detection was conducted with an ECL (Advansta, CA, USA), and results were recorded with ChemiDoc™ XRS^+^ System (Bio-Rad, Hercules, CA, USA).

### 2.8. Protein Identification

Protein bands on SDS-PAGE gels were visualized by silver staining using a SilverQuest Silver Staining kit (Invitrogen, Carlsbad, CA, USA; catalog number LC6070). Bands of interest were excised, digested with sequencing-grade modified porcine trypsin (Promega Corporation, Madison, WI, USA) overnight at 37°C, and analyzed by mass spectrometry on an LC-20AD nanoHPLC-MS/MS (Shimadzu Scientific Instruments) in the Beijing Genomics Institute (Beijing, CHN). A mass tolerance of 20 ppm was permitted for intact peptide masses and 0.6 Da for fragmented ions, with allowance for one missed cleavage in the trypsin digest. Potential variable modifications were Gln to pyro-Glu (N-term Q), oxidation (M), and deamidation (NQ), and the fixed modification was carbamidomethyl (C). Mascot software was used to assay the files in the NCBI protein sequence database.

### 2.9. KRT1 Gene Cloning and Expression

The template sequence of human* KRT1* cDNA in a plasmid pCMV6-Entry was purchased from OriGene (Rockville, MD, USA). The region of* KRT1* from exons 1 through 9 was amplified using two primers: forward, 5′-AAT TTA AAG GAA TTC ATG AGT CGA CAG TTT AGT TC-3′, which included an EcoR I cleavage site sequence and the start codon, and reverse, 5′-GTG TTT CCC AAG CTT TCT GGT TAC TCC GGA ATA AG-3′, which included a Hind III cleavage site rather than the stop codon and a six-His coding sequence to enable recombinant purification. After digestion with EcoRI and HindIII, PCR products were cloned into a pET29 vector. Colonies with* KRT1* DNA inserts were selected; these colonies were amplified, and plasmids were isolated using PureLink Quick Plasmid Miniprep Kit (Invitrogen). The construct was confirmed by sequencing. Mutations to the initial KRT1 plasmid yielded constructs encoding full-length recombinant KRT1-WT, KRT1-MU, and KRT1-DEL (Supplementary Table  1 in Supplementary Material available online at https://doi.org/10.1155/2017/8679841).

Recombinant proteins were produced in* E. coli* BL21 (DE3) (Novagen, Billerica, MA, USA). Colonies were selected and grown in liquid LB medium with 50 *μ*g/mL kanamycin. Expression of KRT1 proteins was induced with 1 mM IPTG (Calbiochem, San Diego, CA). After 3 h, bacteria were harvested and washed with 50 mM Tris-HC1 (pH 8.0). Recombinants were purified with a His-Bind purification kit (Novagen) in the presence of 6.0 M urea (Sigma-Aldrich, St. Louis, MO, USA). Protein concentration was determined using the Micro BSA Assay (Pierce, Rockford, IL, USA) using bovine serum albumin as standard. The proteins were 98% pure as determined by SDS-PAGE and Coomassie blue staining.

### 2.10. ELISA for Detection of Antibodies against KRT1

Nunc MaxiSorp 96-well plates (Fisher Scientific, Richardson, TX, USA) were coated with 2.0 *μ*g/mL of the recombinant KRT1-WT, KRT1-MU, or KRT1-DEL in 0.05 M carbonate/bicarbonate buffer (pH 9.6) by incubation at 4°C overnight. Plates were coated with 0.48 *μ*g/mL of His-Tagged Human ATG8/GABARAPL1 protein (Sino Biological Inc., Beijing, CHN) as controls. The coated plates were blocked by incubation with 10% goat serum (Sigma-Aldrich) in PBS at 37°C for 2 h. The plates were then incubated with test serum diluted 1 : 50 in an incubation solution of 10% goat serum in PBS at 4°C overnight. After five washes with PBS, HRP-conjugated goat anti-human antibodies (Jackson Laboratories) diluted 1 : 5,000 were added, and samples were incubated at 37°C for 2 h. After five washes with PBS, 2,2′-azino-bis(3-ethylbenzthiazoline-6-sulphonate) (ABTS, Shanghai Yuanye Biotechnology Co., Shanghai, CHN) was added to the plates, and the reaction was stopped after 30 minutes using 2 M sulfuric acid. Optical density was read using a Stat Fax-2100 microplate reader (Awareness Technology, Inc., Palm City, FL, USA) at 405 nm. Each sample was tested in triplicate, and a median value, from which the goat serum control value was subtracted, was used for analysis. Experiments on each sample were repeated to ensure reproducibility.

### 2.11. KRT1 Genotyping with PCR-SSP

Genomic DNAs were isolated and purified using QIAamp DNA Blood Mini Kits (QIAGEN China (Shanghai) Co., Ltd.). PCR with sequence-specific primers (PCR-SSP) was used to identify the single-nucleotide polymorphism (SNP, rs14024, A/G) and the microsatellite polymorphism with or without 21-nucleotide deletion in exon 9 of the KRT1 gene. The deleted mutant (KRT1-Del) was detected using PCR by assessing the product size from migration in gels. Three sets of primer pairs for KRT1 genotyping were listed in Supplementary Table  2.

### 2.12. Statistical Analysis

Statistical significance between two groups with frequencies of clinical characteristics was determined using a stand chi-square test (GraphPad Prism 6 version, GraphPad Software, Inc. La Jolla, CA. USA). *p* < 0.05 was considered significant.

## 3. Results

### 3.1. Characteristics of AECA Sera from Renal Transplant Patients with Allograft Dysfunction

**Figure 1:**
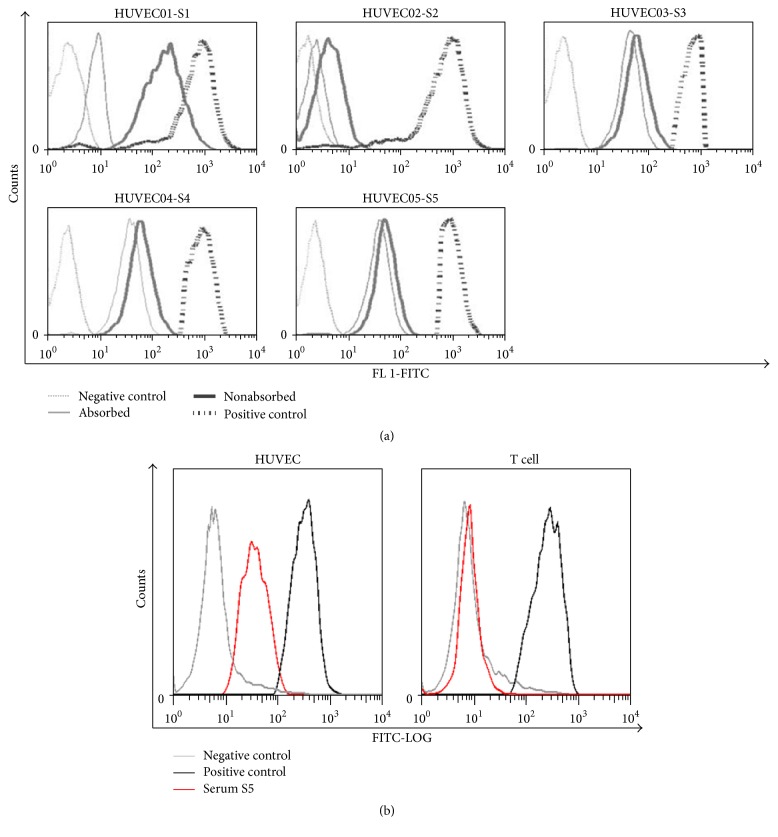
Isolation and identification of AECAs from posttransplant sera. (a) Sera samples from five patients were analyzed by flow cytometry for crossmatches using HUVECs. Normal human serum was used as negative control; HLA-sensitized pooled sera were used as positive control. Profiles before platelet absorption and after platelet absorption are shown. (b) Sample S5 was analyzed by flow cytometry in the presence of HUVEC and cord blood T lymphocytes. The negative control was normal human serum (NC), and the positive control was HLA-sensitized pooled sera.

Serum samples from five patients experiencing renal allograft dysfunction (serum creatinine > 400 *μ*mol/L) and having biopsy-proven rejection with C4d-positive staining or notable glomerulitis and peritubular capillaritis were analyzed (Table 1). Patients S1, S3, S4, and S5 had antibodies against at least one HUVEC sample from a healthy donor as shown by flow cytometry (Figure 1(a)). Patient S1 had anti-HLA-A2 cross-reactive group (CREG) antibodies. After absorption by pooled human platelets, anti-A2 antibodies in serum S1 were not detected by the Luminex assay and no longer bound to HUVECs (Table 1, Figure 1(a)). Although serum S2 had antibodies against DR4, DR7, and DR9 antigens (Table 1), no binding was detected to any of the eight HUVEC samples tested with flow cytometry (Figure 1(a)) and C4d staining was negative. Patient S3 had antibodies against MICA group 1 antigen (MICA-G1), but no anti-HLA antibody was detected in this serum (Table 1). In the serum sample from patient S4, anti-HLA-B5 antibodies were detected. After platelet absorption, no anti-HLA antibodies remained, but its flow crossmatch with HUVCEs remained positive (Figure 1(a)). This serum might have multiple AECAs including antibodies that recognize endothelial cell (EC) surface proteins and anti-B5 CREG antibodies. Interestingly, the serum from patient S5 had antibodies that bound to surface antigens of HUVECs but not to lymphocytes from the same donor (Figure 1(b)). This serum had EC-specific antibodies that were not anti-HLA or anti-MICA antibodies.

### 3.2. Capture of AECA Target Antigens with Immunoprecipitation

**Figure 2:**
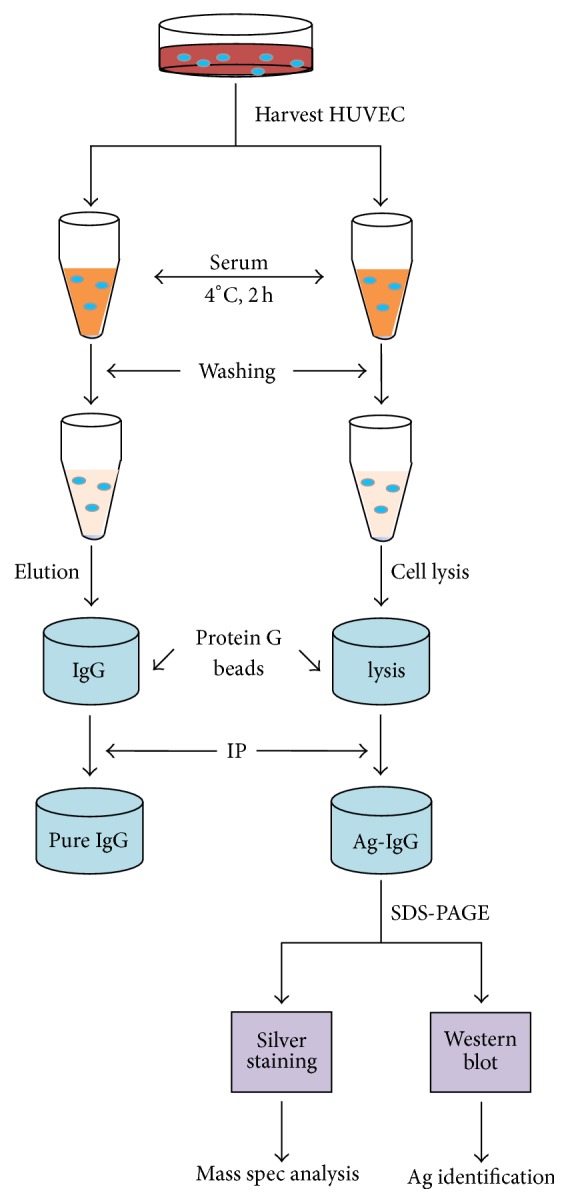
Schematic of procedure used for AECA antibody purification and antigen immunoprecipitation.

**Figure 3:**
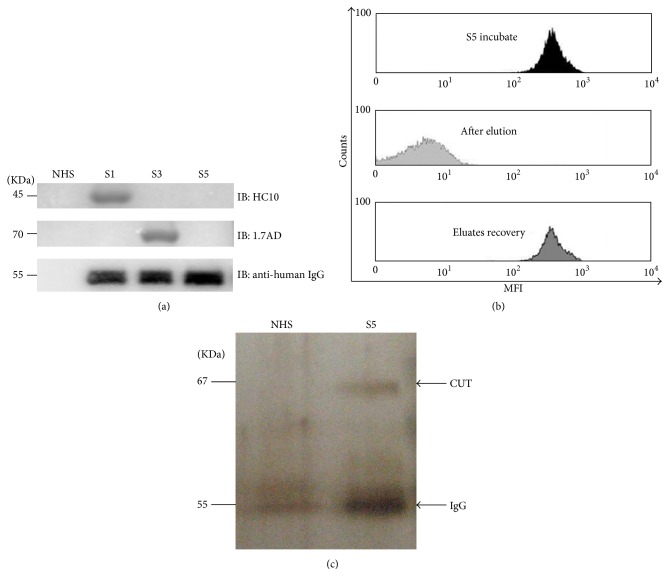
Antibody elution and EC target antigen immunoprecipitation. (a) Sera S1, S3, and S5 with anti-HLA, anti-MICA, and anti-EC antibodies, respectively, as well as normal human serum were incubated with HUVECs. HUVECs were lysed, and the antibody-antigen complex was captured using protein-G magnet beads. Precipitated proteins were separated on SDS-PAGE, and immunoblots (IB) were performed using HC10 (anti-HLA heavy chain), 1.7AD (anti-MICA), and anti-human IgG, respectively. (b) HUVECs were incubated with serum S5 (top), supernatant after elution step (middle), and serum antibodies were enriched by antibody absorption and elution as shown in Figure 2, left. (c) Proteins precipitated with S5 were separated on SDS-PAGE and revealed by silver staining. Normal human serum was used as negative control. The captured antigen from HUVECs and IgG bands are indicated.

To identify AECA target antigens, the updated procedure shown in Figure 2 was used. Serum was incubated with HUVECs. To identify the antibodies that adhered to the surface of the HUVECs, other components of serum were removed by a series of washes. Either the IgG antibodies that bound to the ECs were eluted by acid buffer, followed neutralization with base buffer, and concentrated by protein-G magnet beads, or the antigen/antibody complexes were captured after cell lysis using protein-G magnetic beads. To validate the process, sample S1, which had anti-HLA antibodies (anti-A2), and sample S3 with anti-MICA antibodies (MICA-G1) detected by the Luminex assay were evaluated. Both sera were used for immunoprecipitation and the cell surface target antigens were successfully captured as demonstrated in western blot assays with the specific monoclonal antibodies HC10 and 1.7AD (Figure 3(a)). Sample S5, which had anti-EC-specific antibodies targeting HUVECs, was used for antibody enrichment with antibody absorption and elution (Figure 3(b)). In lysate of sample S5, there were no bands detected with anti-HLA (HC10) or anti-MICA (1.7AD) antibodies, although human IgG globulins were detected like samples S1 and S3 (Figure 3(a)). These results indicated that the captured antigens were EC-specific antigens other than HLA or MICA. In this experiment, the antigen captured by immunoprecipitation from S5 migrated at a molecule weight (MW) of 70 kDa in SDS-PAGE (Large band, Figure 3(c)). The small band at approximately 55 kDa was probably of the IgG heavy chain; therefore, only the band at 70 kDa was taken for further analysis.

### 3.3. KRT1 Is an Important Target of Anti-EC Antibodies

The protein captured by the antibody in sample S5 with MW of approximately 70 was isolated and analyzed by mass spectrometry. The protein with the highest score of 11578.08 was KRT1 with an actual molecular weight 67 kDa (Table 2). Proteins with lower scores and without molecular weight matches are listed in Table 2. In analysis of peptide amino sequences, 315 of 2116 peptide signals (14.89%) were indicative of KRT1, and identified peptides covered 64% of the KRT1 molecule (Supplementary Table  3, Supplementary Figure  1).

**Figure 4:**
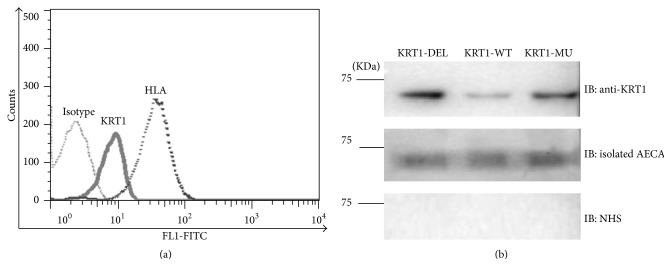
Confirmation of KRT1 as the target of patient antibody. (a) KRT1 was detected on the surface of endothelial cells using a monoclonal antibody; isotype and mAb w6/32 (anti-HLA-class I) were detected as controls. (b) In a western blot, three recombinant KRT1 variants were recognized (IB) by anti-KRT1 polyclonal rabbit serum (top) and by antibodies isolated from serum S5 (middle); normal human serum (NHS) did not react with recombinant KRT1 variants (bottom).

To confirm that patient antibodies recognized KRT1, we first sought to show that KRT1 is expressed on the surface of HUVECs. Using a KRT1-specific monoclonal antibody C2562, we detected KRT1 expression on the surface of HUVECs using flow cytometry (Figure 4(a)). To confirm the result of IP and mass spectrometry analysis, we produced three recombinant proteins known to be encoded by different alleles of* KRT1*. Each of the three KRT1 polymorphic proteins was expressed in bacterial cells. The isolated proteins were recognized in a western blot as bands with molecular weights of 67 kDa using commercial anti-KRT1 polyclonal antibody (Figure 4(b)). The eluate from S5 serum preparation also bound all three polymorphic KRT1 recombinants, whereas normal human serum did not.

### 3.4. Incidence of KRT1-Specific Antibodies in AECA Serum Samples

**Figure 5:**
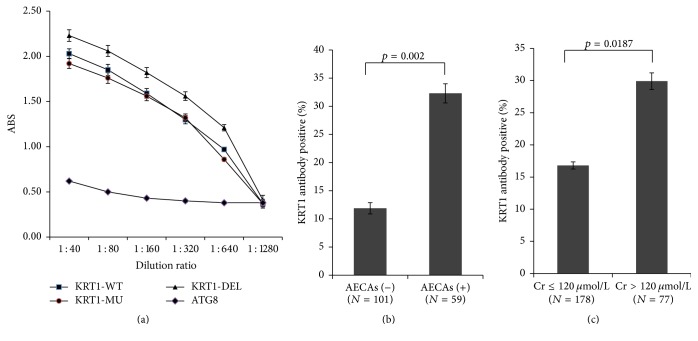
Incidence of KRT1-specific antibodies in kidney transplant recipient serum samples. (a) Serum samples were analyzed using ELISAs for three KRT1 recombinants (KRT1-WT, KRT1-MU, and KRT1-DEL) and ATG8/GABARAPL1. The absorbance at 405 nm is plotted as a function of concentration of serum S5. (b) Percentages of KRT1-positive recipients in AECA-positive and AECA-negative groups. (c) Percentages of KRT1-positive recipients in subjects with serum creatinine level ≤ 120 *μ*mol/L and subjects with serum creatinine level > 120 *μ*mol/L. The statistic *p* values are given.

The three recombinant proteins KRT1-WT, KRT1-MU, and KRT1-DEL and one unrelated protein were used in an ELISA panel to detect KRT1 antibodies. The validation experiments used serum S5. Serum samples prepared by dilution from 1 : 40 to 1 : 1280 were tested. The absorbance decreased as the concentration of serum decreased for KRT1 variants; a dose-response was not observed in the control antigens (ATG8) ELISA (Figure 5(a)). These results validated that the ELISA was sensitive for the detection of serum KRT1 antibodies.

During follow-up of the front 160 recipients of renal allografts, serum samples were obtained. These samples were initially tested for AECA reaction with nondonor HUVECs using flow cytometry and then the same samples were tested for binding to KRT1 using the ELISA. Of the 160 recipients, 59 (36.9%) had AECAs in their posttransplant sera. Of these 59 AECA-positive subjects, 19 (32.2%) had anti-KRT1 antibody IgG. Furthermore, of the 101 recipients who were AECA negative, 12 (11.9%) had antibodies that reacted with KRTI in the ELISA. KRT1-IgG was more frequently detected in the patient group with AECAs than in the AECA-negative group (*x*^2^ = 9.847, *p* = 0.002) (Figure 5(b)).

*KRT1* genotyping was performed on 28 subjects who had high titers of anti-KRT1 antibodies using a sequence-specific primer PCR (SSP-PCR) method. We compared the* KRT1* allele type to antibody preference for KRT1-WT, KRT1-MU, and KRT1-DEL. Analyzing the pattern of antibody reaction, there were 42.86% (12/28) of recipients with anti-KRT1 antibodies to all three KRT1 proteins tested and another 42.86% of recipients with antibodies against KRT1-WT and KRT1-MU. In addition, 2 patients with antibodies were against KRT1-MU and KRT1-DEL, and 2 patients with antibodies only against KRT1-MU. Comparison with antibody serological patterns and patient's self KRT1 genotypes, the results showed that 89% of patients produced anti-KRT1 antibodies reacting with self KRT1 antigens (Table 3). This indicates that KRT1 antibodies are most likely autoantibodies.

To bring this study full circle, we performed a preliminary analysis of the clinical outcomes for kidney transplanted recipients. We analyzed sera from 255 recipients with the KRT1-specific ELISA. In total, we found that 53 recipients (20.8%) had KRT1-IgG against one or more KRT1 allele-specific antigens. Seventy-seven recipients had serum creatinine > 120 *μ*mol/L, indicative of abnormal kidney function. Of these, 29.9% (23/77) had KRT1 antibodies. In the recipient group with serum creatinine ≤ 120, 16.9% (30/178) had KRT1 antibodies (Figure 5(c)). These results suggest that the presence of KRT1 antibodies is significantly associated with deterioration of kidney allograft function (*x*^2^ = 5.531, *p* = 0.0187).

## 4. Discussion

In this study, an immunoprecipitation approach was used to identify a novel antigenic target of anti-endothelial cell antibodies associated with renal allograft dysfunction. Although AECAs are frequently detected [15–17], an effective approach for identification of the antigens on the surface of endothelial cells has been lacking, and routine testing for AECAs has not been implemented because the use of cell-based crossmatch methods has been problematic. We utilized a strategy of antibody absorption and immunoprecipitation to enable identification of target antigens on ECs using mass spectroscopy. KRT1 was identified as a target antigen of AECA from serum of a subject experiencing renal graft rejection. KRT1 is a component of keratin, a fibrous structural complex composed of equal molar amounts of KRT1 and Keratin 10. Keratin is found mainly in skin [30] but is also observed in endothelial cells [25]. In endothelial cells, KRT1 is involved in the lectin complement pathway triggered by oxidative stress [31]. Moreover, KRT1 is polymorphic [24].

Using KRT1 recombinants as target antigens, we developed a KRT1-specific ELISA and used this assay to test our cohort of renal transplant recipients for anti-KRT1 antibodies. In our 255 subjects, the KRT1 antibodies were more in AECA-positive patients than that in AECA-negative group (32.2% versus 11.9%, *p* = 0.002). Anti-KRT1 antibodies may be an important component of non-HLA AECAs in the patients with organ transplantation as KRT1 antibodies were detected in 20.8% of our cohort and were more frequently detected in recipients at risk of transplant failure (*p* = 0.0187). Although the positive rate of KRT1 antibody in the recipients with serum creatinine > 120 *µ*mol/L was significantly higher than that with serum creatinine ≤ 120 *µ*mol/L, the influence the donor-specific antibodies (DSA) against HLA and MICA antigens in the recipients could not be excluded. This limitation in our study can be resolved by expending new transplant cases with available DNA and serum samples. We found that some patients had antibodies against KRT1 before they received an organ transplant. Since the number of these patients was too small, it is difficult to determine whether presence of KRT1 antibodies before transplantation was an increase of transplant rejection. The results with the ELISA assay with these three antigens showed fairly good reproducibility; however, it was clear that some normal human sera had IgG that bound to KRT1. These results support that the antibodies against Keratin 1 are autoantibodies. More work is needed to understand whether these autoantibodies are harmful to patient organs and tissues.

Because KRT1 is polymorphic and the frequencies of the different alleles vary in different populations [24], we investigated the allele-specificity of anti-KRT1 antibodies. From analysis of* KRT1* SNP frequencies [24], we observed that three were common; we refer to the proteins encoded by these three common alleles as KRT1-WT, KRT1-MU, and KRT1-DEL. Using recombinants of these proteins, we showed that most patients had antibodies to the protein encoded by their self-*KRT1* allele. This is a simple but effective method of distinguishing autoantibodies from alloantibodies. There may be instances when an apparent monomorphic pattern is produced by an additive effect of several polymorphic antibodies. It is also possible that relatively weak antibodies could give a false pattern of alloreactivity. In practice, we selected antibodies that were relatively strong, and false patterns have not been a problem. Both types of antibodies may be of interest in the study of transplant failure. Several groups of investigators have now shown that autoantibodies are commonly produced in the course of allograft rejection [8, 32–34]. The probable role of some autoantibodies in the rejection process, such as anti-AT1R antibodies, was recently discussed by others [11–13].

The method described in the present paper was able to identify antibodies in the serum of patients after rejection of an allograft. Immunoprecipitation provided the means of physically isolating the antigenic target from cells. Various AECAs known to recognize cell surface antigens, such as HLA and MICA antigens, were successfully captured using this approach. The AECAs in serum S5 were non-HLA antibodies, and therefore we concentrated our efforts on this sample. We captured antibody-antigen complexes from lysates of endothelial cells, isolated protein by SDS-PAGE, and identified KRT1 by mass spectrometry analysis.

Pretransplant AECA detection in recipients with donor-specific antibodies to HLA does identify those at higher risk for allograft rejection. Our results suggested that the presence of KRT1-IgG also is strongly associated with renal allograft rejection (*p* = 0.0187). Given the increased incidence of allograft rejection in KRT1-antibody-positive cohort, it is worthy to perform additional studies evaluating the mechanism of allograft injury in presence of these antibodies in recipients. Moreover, further studies in larger clinical samples may confirm that these autoantibodies have a role in autodiseases and organ transplant rejection.

## 5. Conclusion

In summary, this study established a more efficient approach to isolate and purify the specific IgG antibodies targeting vascular endothelium antigens using serum samples from the recipients under renal transplant rejection. KRT1 as the target proteins was frequently identified in our experiments with immunoprecipitation and the mass spectrometry. In this article, we first characterize the anti-KRT1 antibodies in kidney transplant patients and the association of anti-KRT1 antibodies with the outcome of allograft function in clinic. In our preliminary investigation, anti-KRT1 antibodies are most likely autoantibodies. A better understanding of these antibodies will be useful to improve long-term allograft survival and benefit the autodisease treatment.

## Supplementary Material

Supplementary Table 1 provides the three full-length amino acid sequences of Keratin 1 alleles and the His-tag sequences for protein purification; Supplementary Table 2 provides the information of primer sequences for Keratin 1 genotyping (PCR-SSP); Supplementary Table 3 indicates 38 peptides of Keratin 1 which were detected in IP and mass spectrometry, moreover, Supplementary Figure 1 depicts the coverage of the identified peptides among Keratin 1 sequence.

## Figures and Tables

**Table 1:** Characteristics of selected sera from patients with kidney allografts undergoing rejection.

Patient serum (number)	Age (year)	Sex	Donor type^*∗*^	Tx (years)	Serum cr. (umol/L)	C4d	Anti-HLA-I	Anti-HLA-II	Anti-MICA	Anti-HUVEC	Anti-EC (platelets^*∗∗*^ absorbed)
s1	46	F	LRD	7	561	+	A2	−	−	+	−
s2	50	F	LRD	5	462	−	−	DR4,7,9	−	−	−
s3	41	M	CAD	10	639	+	−	−	MICA-G1	+	+
s4	42	F	CAD	10	572	+	B5	−	−	+	+
s5	51	M	CAD	0.5	974	+	−	−	−	+	+

^*∗*^CAD: cadaveric donor; LRD: living related donor; ^*∗∗*^pooled multiple human platelets were used to remove anti-HLA-I antibodies from serum.

**Table 2:** The proteins with high score derived from the mass spectrometry analysis.

Protein ID	Description	Protein score	Protein mass (kDa)
tr|H6VRF8|H6VRF8_HUMAN	Keratin 1	11578.08	66.18
sp|P01023|A2MG_HUMAN	Alpha-2-macroglobulin	1526.79	164.61
sp|P01024|CO3_HUMAN	Complement C3	1278.64	188.60
sp|P01009|A1AT_HUMAN	Alpha-1-antitrypsin	793.88	46.88
sp|P15924|DESP_HUMAN	Desmoplakin	695.10	334.02
tr|A6XGL1|A6XGL1_HUMAN	Transthyretin	312.72	20.30
tr|B4DNH8|B4DNH8_HUMAN	Annexin	97.19	21.82
tr|B4DWK8|B4DWK8_HUMAN	Catalase	78.55	53.48
tr|C9JEV0|C9JEV0_HUMAN	Zinc-alpha-2-glycoprotein	66.71	26.50
tr|V9GYE3|V9GYE3_HUMAN	Apolipoprotein A-II	58.66	5.87
sp|P59665|DEF1_HUMAN	Neutrophil defensin 1	47.70	10.54

**Table 3:** Comparison of antibodies patterns and patient's self *KRT1* genotypes.

	Antibodies			Patient		Self-*KRT1* genotypes	
	Anti-WT	Anti-MU	Anti-Del	Number	%	Number	%
1	+	+	+	12	42.86	12	100.00
2	+	+	−	12	42.86	10	83.00
3	−	+	+	2	7.14	2	100.00
4	−	+	−	2	7.14	1	50.00

				28	100.00	25	89.30
