# Supplementary material for: Analysis of Sera of Recipients with Allograft Rejection Indicates That Keratin 1 Is the Target of Anti-Endothelial Antibodies
Source: J Immunol Res. 2017 Feb 7;2017:8679841. doi: 10.1155/2017/8679841 (PMC5318619; doi:10.1155/2017/8679841)
Supplement: Supplementary file 1 — Supplementary Table 1 provides the three full-length amino acid sequences of Keratin 1 alleles and the His-tag sequences for protein purification; Supplementary Table 2 provides the information of primer sequences for Keratin 1 genotyping (PCR-SSP); Supplementary Table 3 indicates 38 peptides of Keratin 1 which were detected in IP and mass spectrometry, moreover, Supplementary Figure 1 depicts the coverage of the identified peptides among Keratin 1 sequence. [file 8679841.f1.pdf]

MSRQFTSRSGYRSGGGFSSGSAGIINYQRRTTSSSTRRSGGGGGRFSSCGGGG
GSFGAGGGFGSRSLVNLGGSKSISISVARGGGRGSGFGGGYGGGGFGGGGFG
GGGFGGGGIGGGGFGGFGSGGGGFGGGGFGGGGYGGGYGPVCPPGGIQEVT
INQSLLQPLNVEIDPEIQKVKSREREQIKSLNNQFASFIDKVRFLEQQNQVLQT
KWELLQQVDTSTRTHNLEPYFESFINNLRRRVDQLKSDQSRLDSELKNMQDM
VEDYRNKYEDEINKRTNAENEFVTIKKDVDGAYMTKVDLQAKLDNLQQEID
FLTALYQAELSQMQTQISETNVILSMDNNRSLDLDSIIAEVKAQYEDIAQKSKA
EAESLYQSKYEELQITAGRHGDSVRNSKIEISELNRVIQRLRSEIDNVKKQISNL
QQSISDAEQRGENALKDAKNKLNDLEDALQQAKEDLARLLRDYQELMNTKL
ALDLEIATYRTLLEGEESRMSGECAPNVSVSVSTSHTTISGGGSRGGGGGGYG
SGGSSYGSGGGSYGSGGGGGGGRGSYGSGGSSYGSGGGSYGSGGGGGGHGS
YGSGSSSGGYRGGSGGGGGSSGGRGSGGGSSGGSIGGRGSSSGGVKSSGGS
SSVKFVSTTYSGVTR

1-5	11-15	21-25	31-35
6-10	16-20	26-30	

Supplementary Figure 1: Coverage of keratin 1 associated peptides identified by Mass Spectrometry in Keratin 1.

Number indicates the frequency of the detected peptide tagged with corresponding color.

Supplementary Table 1: Amino acid of keratin-1 recombinants.

Name	Amino acid sequence
**KRT1-WT**	MSRQFSSRSGYRSGGGFSSGSAGIINYQRRTTSSSTRRSGGGGGRFSSCGGGGGSFGAGGGFGSRSLVNLGGSKSISISVARGGGRGSGFGG<br>GYGGGGFGGGGFGGGGFGGGGIGGGGFGGFGSGGGGFGGGGFGGGGYGGGYGPVCPPGGIQEVTINQSLLQPLNVEIDPEIQKVKSRER<br>EQIKSLNNQFASFIDKVRFLEQQNQVLQTKWELLQQVDTSTRTHNLEPYFESFINNLRRRVDQLKSDQSRLDSELKNMQDMVEDYRNKYE<br>DEINKRTNAENEFVTIKKDVDGAYMTKVDLQAKLDNLQQEIDFLTALYQAELSQMQTQISETNVILSMDNNRSLDLDSIIAEVKAQYEDIA<br>QKSKAEAESLYQSKYEELQITAGRHGDSVRNSKIEISELNRVIQRLRSEIDNVKKQISNLQQSISDAEQRGENALKDAKNKLNDLEDALQQ<br>AKEDLARLLRDYQELMNTKLALDLEIATYRTLLEGEESRMSGECAPNVSVSVSTSHTTISGGGSRGGGGGGYGSGGSSYGSGGGSYGSGG<br>GGGGGRGSYGSGGSSYGSGGGSYGSGGGGGGHGSYGSGSSSGGYRGGSGGGGGGSSGGRGSGGGSSGGSIGGRGSSSGGVKSSGGSSSV<br>KFVSTTYSGVTR*KLAAALEHHHHHH*
**KRT1-MT**	MSRQFSSRSGYRSGGGFSSGSAGIINYQRRTTSSSTRRSGGGGGRFSSCGGGGGSFGAGGGFGSRSLVNLGGSKSISISVARGGGRGSGFGG<br>GYGGGGFGGGGFGGGGFGGGGIGGGGFGGFGSGGGGFGGGGFGGGGYGGGYGPVCPPGGIQEVTINQSLLQPLNVEIDPEIQKVKSRER<br>EQIKSLNNQFASFIDKVRFLEQQNQVLQTKWELLQQVDTSTRTHNLEPYFESFINNLRRRVDQLKSDQSRLDSELKNMQDMVEDYRNKYE<br>DEINKRTNAENEFVTIKKDVDGAYMTKVDLQAKLDNLQQEIDFLTALYQAELSQMQTQISETNVILSMDNNRSLDLDSIIAEVKAQYEDIA<br>QKSKAEAESLYQSKYEELQITAGRHGDSVRNSKIEISELNRVIQRLRSEIDNVKKQISNLQQSISDAEQRGENALKDAKNKLNDLEDALQQ<br>AKEDLARLLRDYQELMNTKLALDLEIATYRTLLEGEESRMSGECAPNVSVSVSTSHTTISGGGSRGGGGGGYGSGGSSYGSGGGSYGSGG<br>GGGGGRGSYGSGGSSYGSGGGSYGSGGGGGGHGSYGSGSSSGGYRGGSGGGGGGSSGGRGSGGGSSGGSIGGRGSSSGGVKSSGGSSSV<br>RFVSTTYSGVTR*KLAAALEHHHHHH*
**KRT1-DEL**	MSRQFSSRSGYRSGGGFSSGSAGIINYQRRTTSSSTRRSGGGGGRFSSCGGGGGSFGAGGGFGSRSLVNLGGSKSISISVARGGGRGSGFGG<br>GYGGGGFGGGGFGGGGFGGGGIGGGGFGGFGSGGGGFGGGGFGGGGYGGGYGPVCPPGGIQEVTINQSLLQPLNVEIDPEIQKVKSRER<br>EQIKSLNNQFASFIDKVRFLEQQNQVLQTKWELLQQVDTSTRTHNLEPYFESFINNLRRRVDQLKSDQSRLDSELKNMQDMVEDYRNKYE<br>DEINKRTNAENEFVTIKKDVDGAYMTKVDLQAKLDNLQQEIDFLTALYQAELSQMQTQISETNVILSMDNNRSLDLDSIIAEVKAQYEDIA<br>QKSKAEAESLYQSKYEELQITAGRHGDSVRNSKIEISELNRVIQRLRSEIDNVKKQISNLQQSISDAEQRGENALKDAKNKLNDLEDALQQ<br>AKEDLARLLRDYQELMNTKLALDLEIATYRTLLEGEESRMSGECAPNVSVSVSTSHTTISGGGSRGGGGGGYGSGGSSYGSGGGSYGSGG<br>GGGGGRGSY-------------GSGGGSYGSGGGGGGHGSYGSGSSSGGYRGGSGGGGGGSSGGRGSGGGSSGGSIGGRGSSSGGVKSSGGSSSV<br>KFVSTTYSGVTR*KLAAALEHHHHHH*

‘**---**’indicate the amino acid deleted; ‘KLAAALEHHHHHH’ is specially designed tag for recombinant protein purification.

Supplementary Table 2. Primers used for Genotyping.

Name	Sequence	Location	Tm
rs14024	F:5’-CTGTGAGCACAAGCCACACC-3’	4791-4810	**59.4**
	R: 5’-GGAATAAGTGGTAGAAACAAACT-3’	5178-5200	**52.5**
	R: 5’-GGAATAAGTGGTAGAAACAAACC-3’	5178-5200	**55.0**
rs61226348	F:5’-CTGTGAGCACAAGCCACACC-3’	4791-4810	**59.4**
	R: 5’-TCCGGAGCCGTAGCTGCCATG-3’	5003-5023	**69.9**
GAPDH	F:5’-CCCTTTGAGTTTGATGATGC-3’	1671-1690	**55.4**
	R: 5’-GGAAGATGGTGATGGGATTT-3’	2349-2368	**56.0**

Supplementary Table 3: The keratin1-associated peptide by mass spectrometry.

Peptide Number	Peptide Sequence	Start	End	Frequency	Frequency（%）
1	SGGGFSSGSAGIINYQR	13	29	10	3.17
2	FSSCGGGGGSFGAGGGFGSR	46	65	11	3.49
3	SLVNLGGSKSISISVAR	66	82	3	0.95
4	SLNNQFASFIDK	186	197	14	4.44
5	SLNNQFASFIDKVR	186	199	5	1.59
6	FLEQQNQVLQTK	200	211	23	7.30
7	WELLQQVDTSTR	212	223	6	1.90
8	FLEQQNQVLQTKWELLQQVDTSTR	200	223	2	0.63
9	THNLEPYFESFINNLR	224	239	7	2.22
10	THNLEPYFESFINNLRR	224	240	5	1.59
11	LDSELKNMQDMVEDYR	252	267	5	1.59
12	NMQDMVEDYR	258	267	25	7.94
13	RTNAENEFVTIK	277	288	1	0.32
14	LALDLEIATYR	278	288	10	3.17
15	TNAENEFVTIKK	278	289	24	7.62
16	DVDGAYMTK	290	298	2	0.63
17	DVDGAYMTKVDLQAK	290	304	2	0.63
18	LDNLQQEIDFLTALYQAELSQMQTQISETNVILSMDNNR	305	343	2	0.63
19	SLDLDSIIAEVK	344	355	15	4.76
20	AQYEDIAQK	356	364	4	1.27
21	SKAEAESLYQSK	365	376	9	2.86
22	AEAESLYQSK	367	376	8	2.54
23	AEAESLYQSKYEELQITAGR	367	386	2	0.63
24	YEELQITAGR	377	386	16	5.08
25	IEISELNR	396	403	6	1.90
26	KQISNLQQSISDAEQR	417	432	4	1.27
27	QISNLQQSISDAEQR	418	432	15	4.76
28	QISNLQQSISDAEQRGENALK	418	438	2	0.63
29	NKLNDLEDALQQAK	442	455	6	1.90
30	LNDLEDALQQAK	444	455	4	1.27

31	LNDLEDALQQAKEDLAR	444	460	4	1.27
32	LLRDYQELMNTK	461	472	6	1.90
33	DYQELMNTK	464	472	4	1.27
34	LALDLEIATYR	473	483	8	2.22
35	TLLEGEESR	484	492	5	1.59
36	MSGECAPNVSVSVSTSHTTISGGGSR	493	518	8	2.54
37	GGGGGGYGSGGSSYGSGGGSYGSGGGGGGGR	519	549	25	7.94
38	GSYGSGGSSYGSGGGSYGSGGGGGGHGSYGSGSSSGGYR	550	588	7	2.22
				315	100
